# The suberin transporter StABCG1 is required for barrier formation in potato leaves

**DOI:** 10.1038/s41598-025-89032-x

**Published:** 2025-03-07

**Authors:** Elvio Henrique Benatto Perino, Ulrike Smolka, Karin Gorzolka, Ramona Grützner, Sylvestre Marillonnet, Khabat Vahabi, Sabine Rosahl

**Affiliations:** 1 Department of Stress and Developmental Biology, Leibniz Institute of Plant Biochemistry, Weinberg 3, 06120 Halle (Saale), Germany; 2 Anhalt University of Applied Sciences, Bernburger Str. 55, 06366 Köthen, Germany; 3 Department of Cell and Metabolic Biology, Leibniz Institute of Plant Biochemistry, Weinberg 3, 06120 Halle (Saale), Germany; 4 Present Address: Science Support Platform/Biotic Interactions2, Leibniz Institute of Vegetable and Ornamental Crops (IGZ) e.V., Theodor-Echtermeyer-Weg 1, 14979 Großbeeren, Germany; 5 Present Address: Bioprocess Engineering, University Hohenheim, Stuttgart, Germany; 6 Present Address: Ecological Chemistry, Plant Analysis and Stored Product Protection, Julius-Kühn-Institute, Berlin, Germany

**Keywords:** Suberin, *Solanum tuberosum*, Potato, Wound healing, Hydroxycinnamic acid amides, Secondary metabolism, Wounding

## Abstract

Suberin is a hydrophobic biopolymer that acts as an internal and external diffusion and transpiration barrier in plants. It is involved in two phases of wound healing, i.e. initial closing layer formation and subsequent wound periderm development. Transcriptomic and metabolomic analyses of wounded potato leaf tissue revealed preferential induction of cell wall modifying processes during closing layer formation, accompanied by a highly active defense response. To address the importance of suberin in this process, we generated loss of function mutants by CRISPR-Cas9 editing the suberin transporter gene *StABCG1*. Both wound-induced *StABCG1* transcript levels and suberin formation around wounded leaf tissue were reduced in CRISPR-lines. Moreover, wound-induced tissue damage was characterized by browning of wound-adjacent areas. Transcriptome analyses of these areas revealed up-regulation of genes encoding defense proteins and enzymes of the phenylpropanoid pathway. Levels of hydroxycinnamic acid amides, acting in defense and in cell wall reinforcement, were drastically enhanced in CRISPR compared to control plants. These results suggest that the reduction in suberin formation around wounded tissue leads to a loss of barrier function, resulting in tissue browning due to enhanced exposure to oxygen.

## Introduction

Suberin is a lipid biopolymer that serves as a diffusion and transpiration barrier at the boundary of tissue layers in plants, such as the exo- and endodermis of roots and the periderm of potato tubers. In addition, suberin is synthesized in response to wounding and pathogen infection and plays a role in sealing the surface of unprotected plant tissues.

Glycerol, ω-hydroxy- and dicarboxylic long-chain fatty acids, long-chain primary alcohols, as well as ferulic acid have been shown to be part of suberin^1^, which consists of covalently linked polyaliphatic and lignin-like polyphenolic domains^2,3^.

Potato tuber periderm is a model system to study suberin synthesis, composition and function. Synthesis of suberin requires the β-ketoacyl-CoA synthase StKCS6^4^, the fatty acid hydroxylase CYP86A33^5^ and a feruloyl transferase StFHT^6^. Mutant analyses suggested that monomers are transported out of the cell by the plasma membrane ABC transporter StABCG1^7^. Reducing the expression of biosynthesis and transporter genes by RNA interference (RNAi) results in tubers that are discolored, have a rough and scabby surface and, importantly, show significant weight loss correlating with their inability to form suberized phellem cells. Metabolite analyses reveal major defects in the suberin composition of the tuber periderm in these plants^4–7^.

In transcriptomic analysis, *StABCG1* was identified as a gene activated in response to treatment with the pathogen-associated molecular pattern (PAMP) Pep-13^7^. Pep-13 is a 13 amino acid oligopeptide derived from a transglutaminase from *Phytophthora*^8^, which elicits salicylic acid- and jasmonic acid-dependent defense responses in potato^9,10^. This PAMP-induced expression of *StABCG1* is in accordance with other correlative data suggesting a role for suberin in pathogen defense^11^.

Suberin formation in response to injury is an important part of the process of wound healing and has been extensively studied in potato tubers^12,13^. The first phase of wound healing is characterized by the formation of a closing layer. This involves the synthesis of phenolic compounds, such as hydroxycinnamic acid amides (HCAAs), which are incorporated into the cell wall and, thus, contribute to the formation of a reinforced boundary^14^. In addition, suberization of existing cells bordering the wound site takes place^15^. In the second phase, a wound periderm consisting of suberized cells is formed from a newly developed phellogen or cork cambium^15^.

To characterize wound healing in leaves, we performed transcriptomic and metabolomic analyses of wounded potato plants. Both approaches revealed the preferential induction of cell wall reinforcement processes, accompanied by a highly expressed defense response. Wounding of suberin-deficient plants, generated by CRISPR-Cas9 editing of the suberin transporter gene *StABCG1*, resulted in reduced wound suberin formation, accompanied by major transcriptomic and metabolomic changes, as well as tissue browning. Thus, wound suberin formation is required for effective barrier formation in potato leaves.

## Results

### Suberin biosynthetic genes are expressed in potato leaves in response to Pep-13 and wounding

**Fig. 1:**
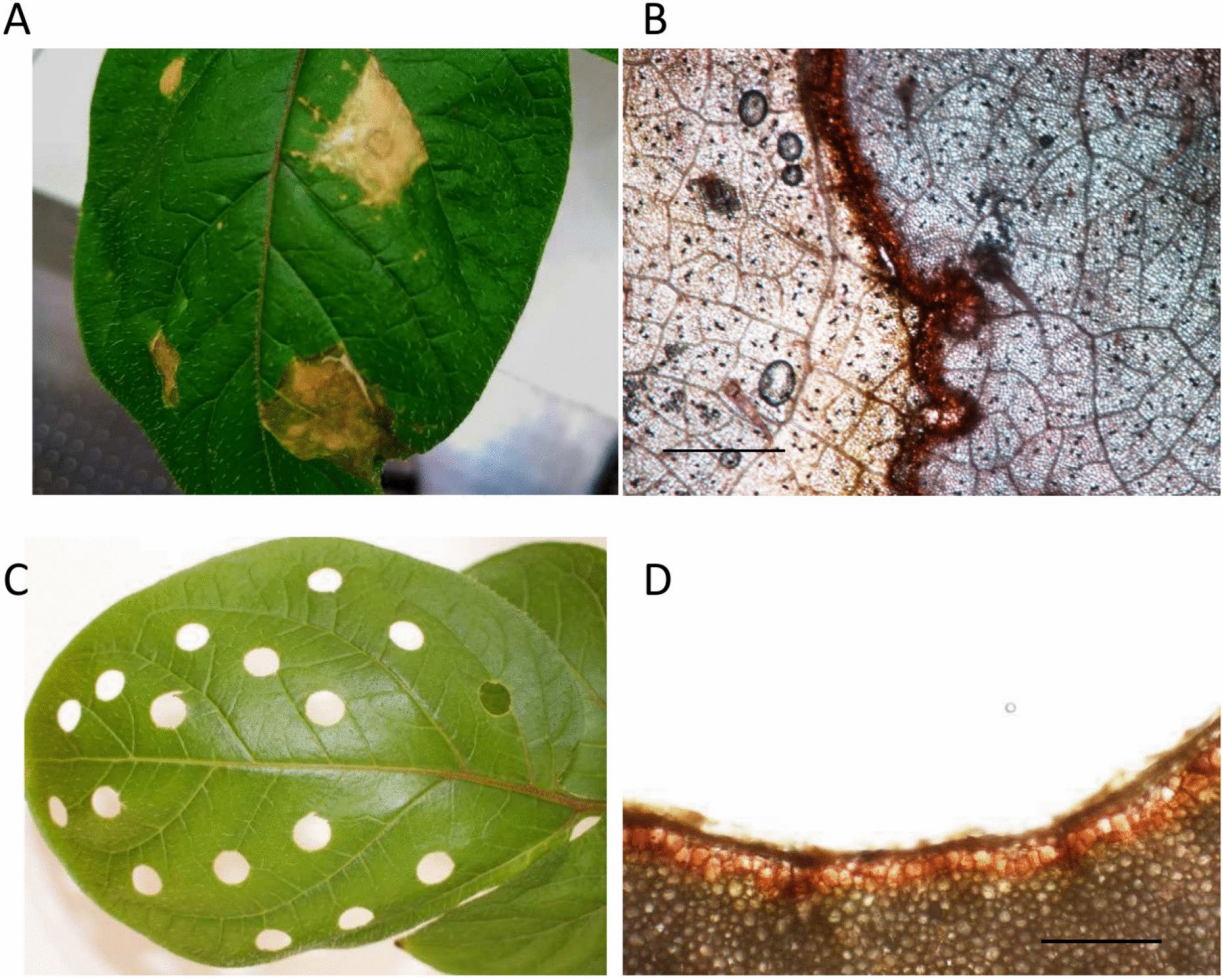
Suberin formation around necrotic tissue in potato leaves. Potato leaves were infiltrated with Pep-13 (**A**, **B**) or wounded with a corkborer (**C**, **D**). Photos were taken 10 days later (**A**, **C**). Suberin was stained with SudanIII (**B**, **D**) and visualized by microscopy. Scale bars represent 1 mm (**B**) and 0.5 mm (**D**).

**Fig. 2:**
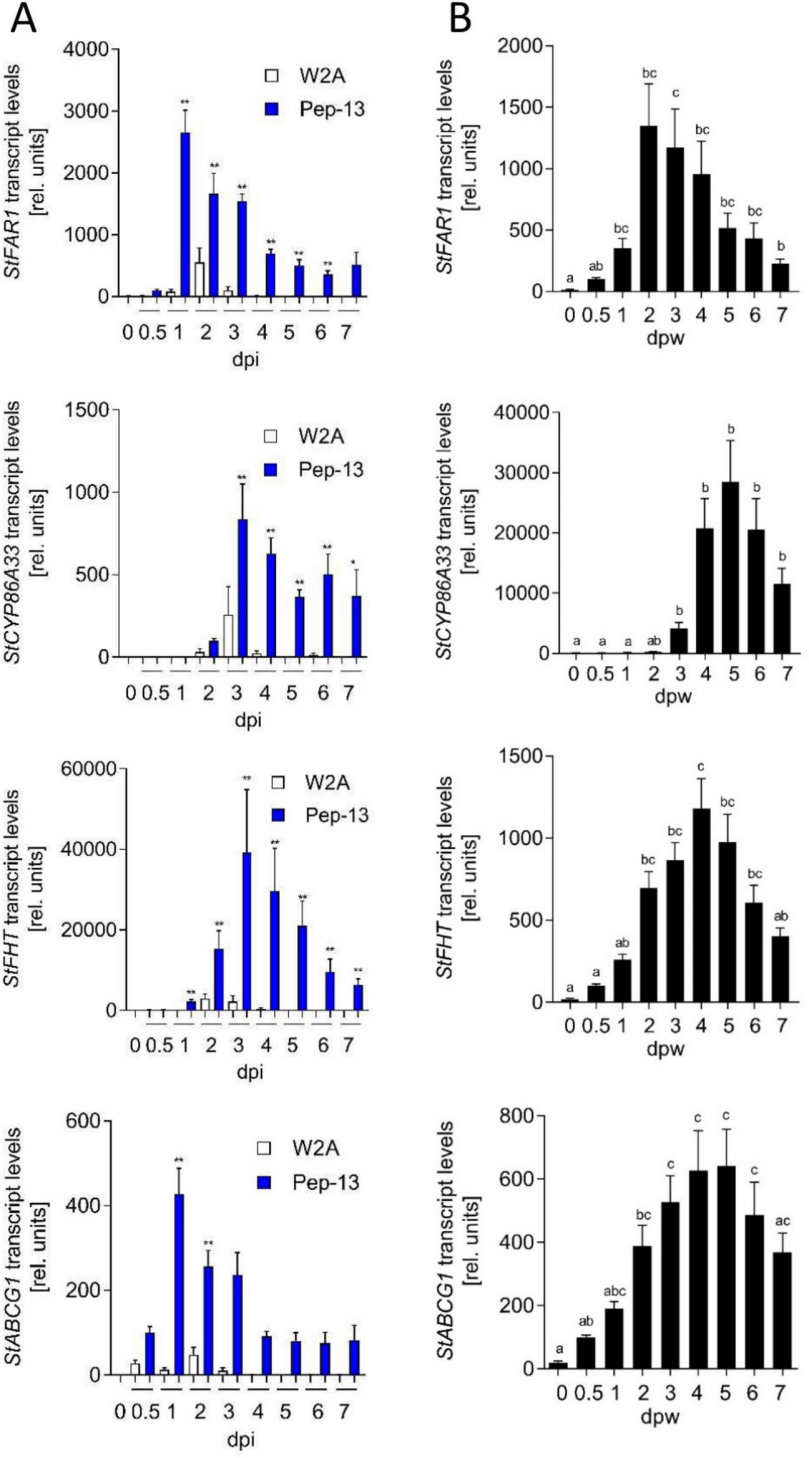
Pep-13- and wound-induced expression of suberin-related genes. RNA was isolated from phytochamber-grown potato plants after infiltration of (**A**) Pep-13 (blue bars) and W2A (white bars) or (**B**) after wounding (black bars) at the time points indicated and subjected to qRT-PCR analyses for expression of *StFAR1*, *StCYP86A33*, *StFHT*, *StABCG1*. Data are derived from three independent experiments (n ≥ 15). Statistical analyses were performed using Mann Whitney U test (A), comparing values to those obtained at 0.5 h, or Kruskal–Wallis test (B). *p < 0.05; **p < 0.01.

The infiltration of Pep-13 into potato leaves leads to the SA- and JA-dependent activation of defense responses, including hypersensitive cell death in the infiltrated area^10^. These Pep-13-induced lesions (Fig. 1A) are surrounded by suberized cell layers, as concluded from Sudan III-staining (Fig. 1B). Similarly, removing disks from potato leaves using a corkborer (Fig. 1C) left damaged tissue with suberized borders (Fig. 1D). We performed qRT-PCR analyses with RNA from Pep-13-infiltrated leaves and wounded leaf tissue and analyzed the expression of genes encoding predicted suberin biosynthesis enzymes, such as *FATTY ACID REDUCTASE1* (*StFAR1*; PGSC0003DMG401007406), *FATTY ACID OMEGA-HYDROXYLASE* (*StCYP86A33*; PGSC0003DMG400030349^5^) and *FERULOYLHYDROXYCINNAMOYL TRANSFERASE* (*StFHT*; PGSC0003DMG400031731^6^), as well as the gene encoding the suberin transporter StABCG1 (PGSC0003DMG400023506^7^). Transcripts of all four genes accumulated both in response to Pep-13 treatment (Fig. 2A), but not after infiltration of the nearly inactive analog W2A^8^, and after wounding (Fig. 2B). The kinetics of *StFAR1* and *StABCG1* transcript accumulation in response to Pep-13 treatment were similar, with the highest levels occurring one day after infiltration, whereas both *StCYP86A33* and *StFHT* transcripts accumulated at later time points (Fig. 2A). After wounding, *StFAR1* transcript accumulation was significantly enhanced after one day, *StFHT* and *StABCG1* after two days and *StCYP86A33* after three days (Fig. 2B). Thus, expression of the four genes analyzed, three of which^5–7^ had been identified as tuber-specific genes, was induced by Pep-13 treatment and wounding.

### Transcriptomic and metabolomics changes in potato leaves in response to wounding

To obtain a broader view of wound-induced changes in transcripts and metabolites, RNA sequencing and untargeted metabolite profiling were performed with wounded leaf tissue. Leaves of phytochamber-grown potato were wounded by a corkborer. Three and seven days after wounding, tissue was obtained using a larger corkborer, covering mostly the wounded and only minimal amounts of the adjacent tissue. RNA isolated from this tissue was subjected to RNA sequencing.

**Fig. 3:**
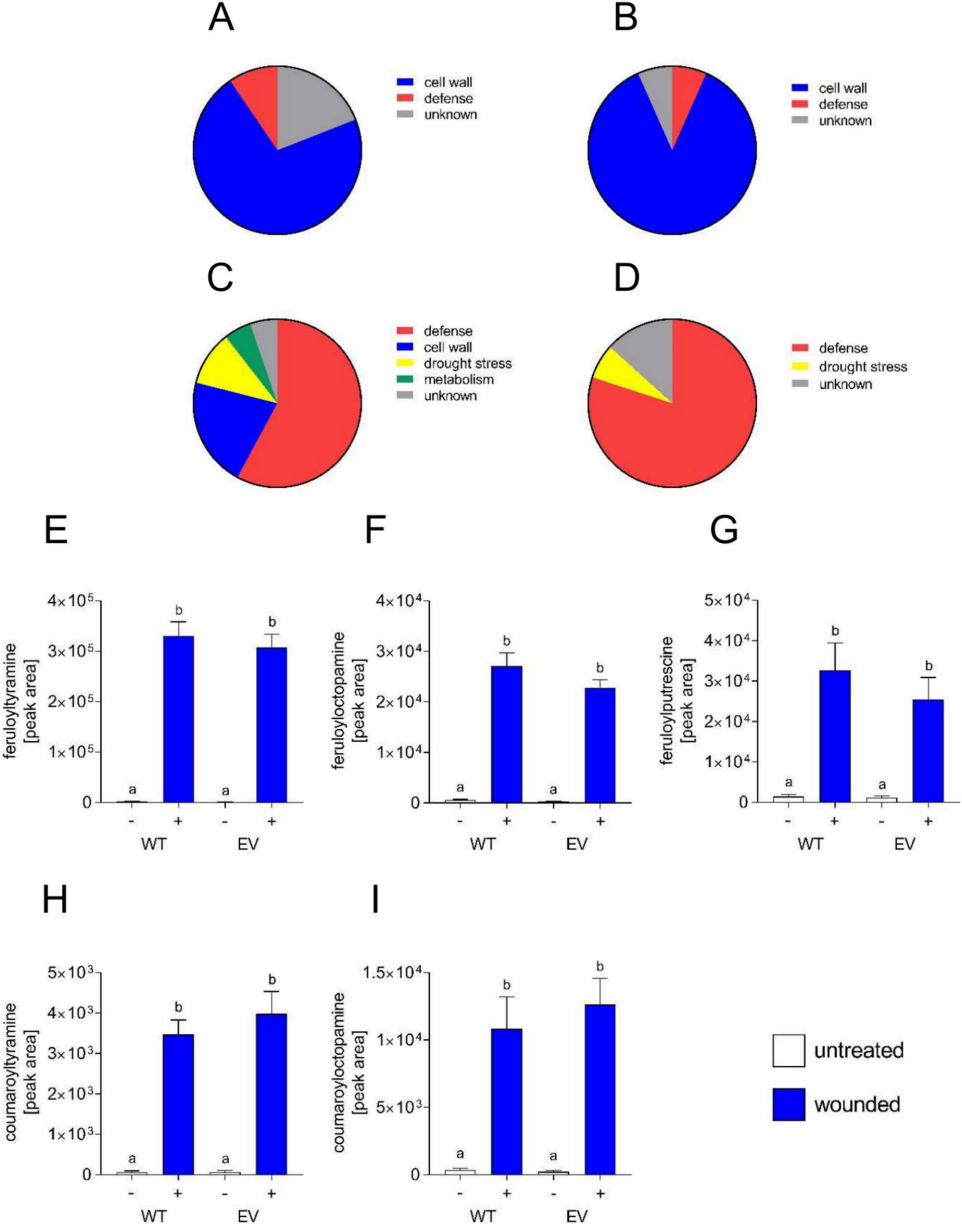
Transcriptomic and metabolomic changes in wounded potato leaves. RNA was isolated from potato leaves 0, 3 (**A**, **C**) and 7 (**B**, **D**) days after wounding and subjected to RNA sequencing. Annotations of genes induced at least 100 fold compared to the 0 time point (**A**: n = 21, **B**: n = 15) as well as the highest expresssed genes (**C**: n = 19, **D**: n = 15) are shown. E-I: Wound-induced accumulation of hydroxycinnamic acid amides. Metabolites were extracted from wild type (WT) or empty vector carrying (EV) potato leaves either untreated (−) or wounded for ten days ( +) and subjected to UPLC-ESI-QTOF-MS analyses. Levels of feruloyltyramine (**E**), feruloyloctopamine (**F**), feruloylputrescine (**G**), coumaroyltyramine (**H**), coumaroyloctopamine (**I**) are shown. Data are derived from three indpendent experiments (n ≥ 20). Statistical analyses were performed using Kruskal–Wallis test.

Three days after wounding, 21 genes were at least 100 fold higher expressed in wounded tissue compared to unwounded tissue. Fifteen of these genes were predicted to encode proteins associated with the modification or strengthening of cell walls, such as peroxidases, laccases and the suberin-associated feruloyl transferase StFHT (Fig. 3A; Supplemental Table S1). Seven days after wounding, 13 out of 15 genes, which were over 100 fold induced in wounded tissue, are predicted to encode proteins associated with cell wall processes (Fig. 3B; Supplemental Table S2), suggesting that fortification of the outer layer of the tissue is the major induced process occurring in wounded leaves.

288 genes were more than 2.5 fold induced in wounded tissue. Among the 19 highest expressed genes in this group, 11 are predicted to encode proteins involved in defense responses, such as PATHOGENESIS-RELATED (PR) 3 and 10 and proteinase inhibitors (Fig. 3C; Supplemental Table S3). Seven days after wounding, 12 of the 14 highest expressed genes encode defense-related genes (Fig. 3D; Supplemental Table S4). Our transcriptome analyses thus revealed that cell wall repair and enforcement mechanisms are the predominantly induced processes, accompanied by a highly expressed defense response.

Untargeted metabolite profiling was performed on tissue obtained as described before. Both wild type plants and transgenic empty vector (EV) plants were analyzed to assess possible differences due to the transformation procedure. About 4000 features were detected in the positive mode and annotation was performed for hydroxycinnamic acid amides. Interestingly, feruloyltyramine was the metabolite that accumulated to the highest levels in wounded tissue of both wild type and EV plants (Fig. 3E), followed by feruloylcotopamine (Fig. 3F) and feruloylputrescine (Fig. 3G). Other HCAAs, such as coumaroyltyramine (Fig. 3H) and coumaroyloctopamine (Fig. 3I) also showed significantly enhanced levels in wounded tissue. These results are in accordance with the proposed role of HCAAs in cell wall reinforcement.

### Generation of CRISPR-Cas9-edited potato plants

**Fig. 4:**
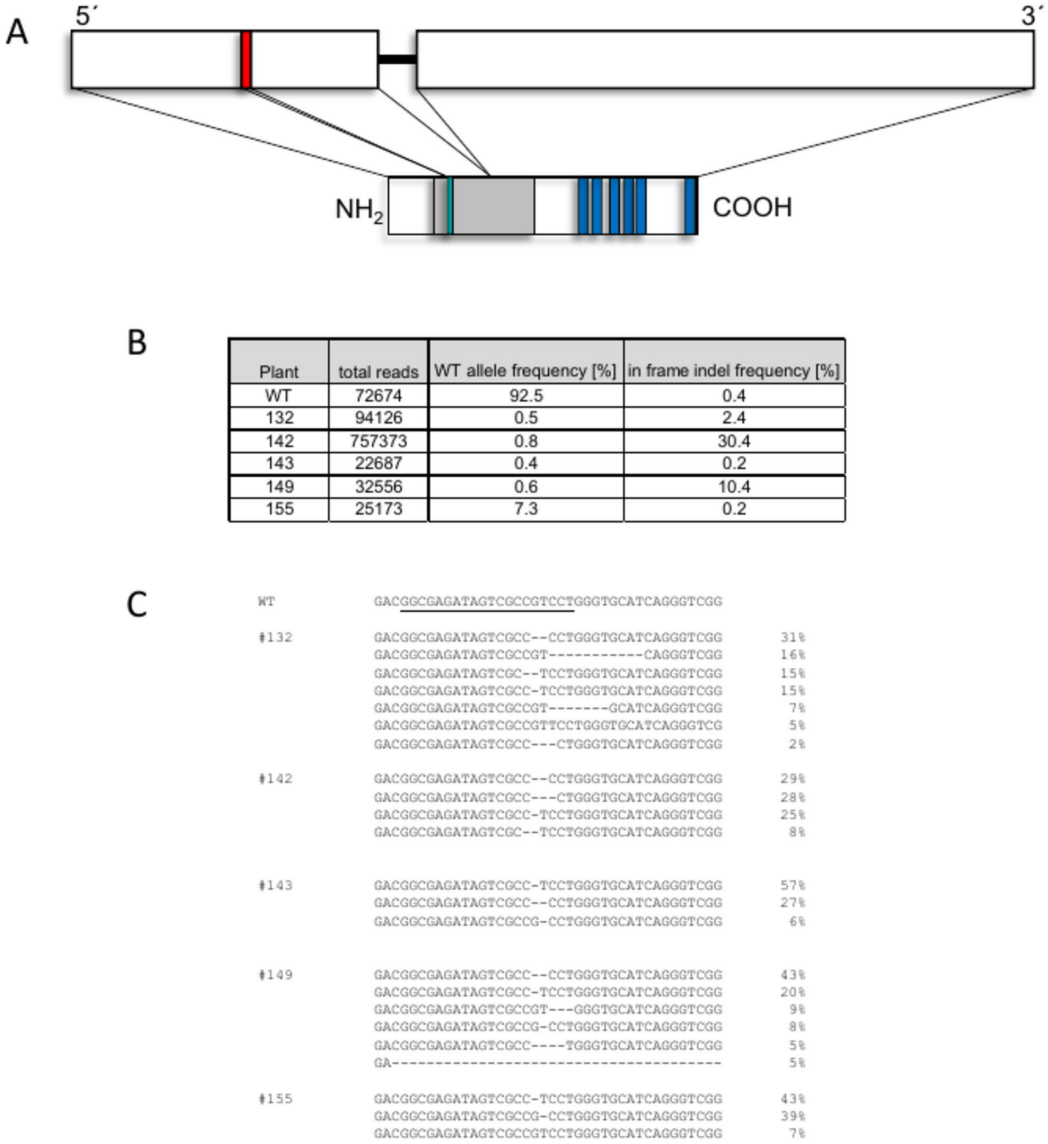
Structure of the *StABCG1* gene/protein and generation of *StABCG1*-CRISPR-edited potato plants. (**A**) Schematic representation of the *StABCG1* gene (upper panel). White boxes: exons, black line: intron. red bar: sequence homologous to the guide RNA. Lower panel: protein structure of StABCG1. Gray box: ABC transporter domain, green box: ATP binding domain, blue boxes: transmembrane domains. (**B**) Percentage of wild type alleles and in-frame indel mutations in *StABCG1*-CRIPSR plants 132, 142, 143, 149 and 155. PCR amplicons derived from genomic DNA of the plants indicated were subjected to Illumina sequencing. (**C**) Nucleotide sequence of edited alleles in *StABCG1*-CRIPSR plants 132, 142, 143, 149 and 155 and percentage of their occurrence. The sequence homologous to the guide RNA is underlined.

To address the role of suberin for wound responses, we edited the *StABCG1* gene encoding a suberin transporter using CRISPR-Cas9. A single guide RNA was designed to target the coding region of the N-terminal part of the protein close to the ATP binding site (Fig. 4A). Binary vectors carrying the Cas9 gene and the guide RNA were transferred to *Agrobacterium tumefaciens*. Subsequent leaf disk transformation of *Solanum tuberosum* L. cv Désirée resulted in the generation of more than 100 independent transformants, which were subsequently analyzed by PCR. Edited plants were preselected by Sanger sequencing of an amplified fragment covering the sgRNA target site. PCR fragments from five plants were subsequently subjected to Illumina sequencing. At least 22.000 reads were obtained for each plant (Fig. 4B). More than four edited alleles of the tetraploid potato were detected in most CRISPR lines, presumably because the primary transformants analyzed contain chimeric tissue due to the continuous activity of Cas9 (Fig. 4C). Plant 142 and 149 had a three base pair deletion each, which would result in the loss of a valine and a leucine, respectively, in the encoded proteins. The same deletion of plant 142 also occurred in plant 132, but at a low percentage (2%). In plant 155, we detected wild type alleles at 7%. Only in plant 143, all alleles detected were edited. Based on these results, plants 143 and also 132 were considered to be fully edited plants.

### Loss of *StABG1* expression correlates with reduced suberin formation

**Fig. 5:**
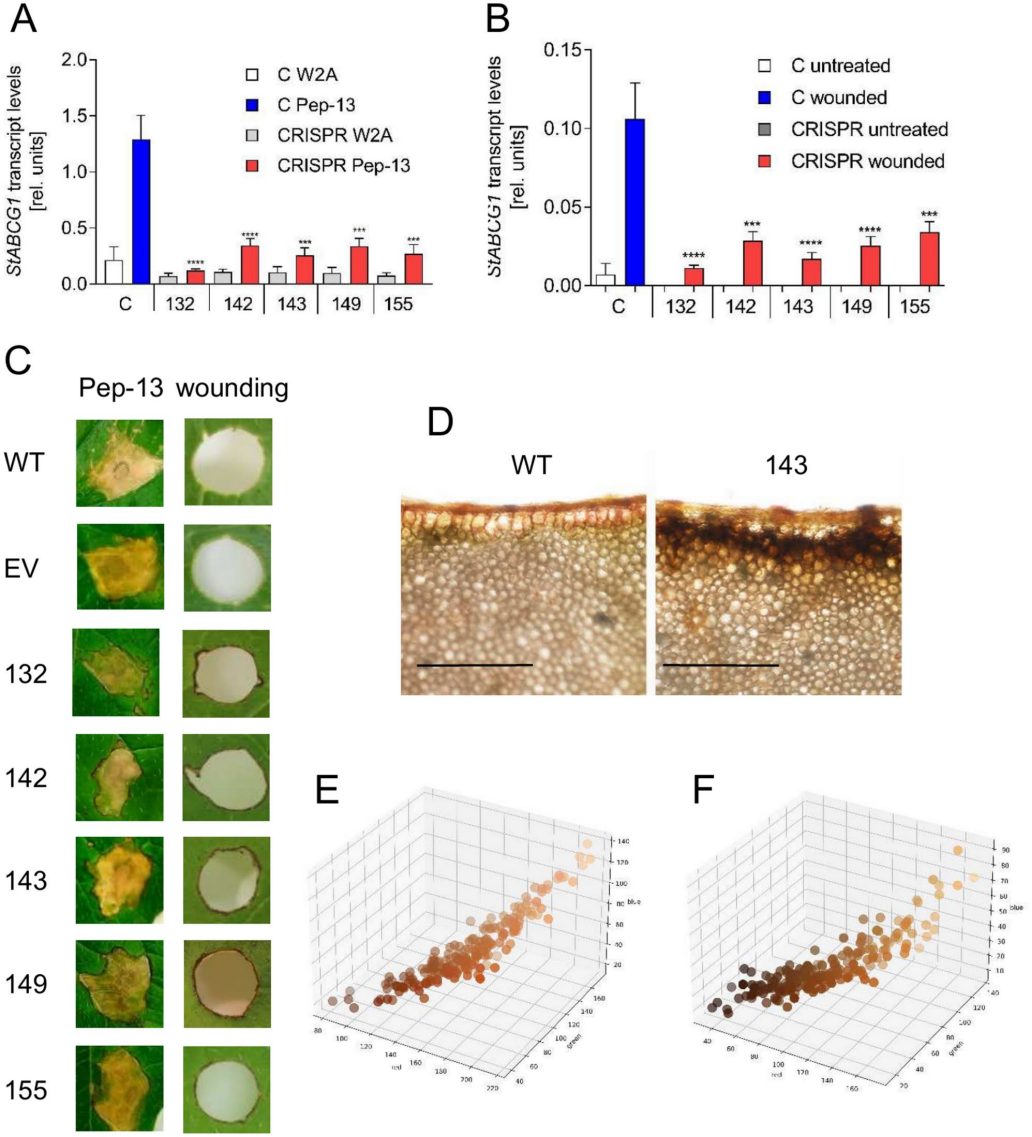
Reduced *StABCG1* expression in *StABCG1*-CRISPR plants correlates with loss of suberin formation and tissue browning. (**A**, **B**) RNA was isolated from phytochamber-grown potato plants (wild type and empty vector carrying plants (“C”) and *StABCG1-*CRISPR-edited plants 132, 142, 143, 149 and 155) 24 h after infiltration of Pep-13 (blue and red bars) or W2A (white and grey bars; **A**) or 0 (white and grey bars) and 3 (blue and red bars) days after wounding (**B**) and subjected to qRT-PCR analyses for expression of *StABCG1.* Data are derived from two independent experiments (n = 8). Statistical analyses were performed using Mann Whitney U test (**p < 0.01; ***p < 0.001). (**C**) Phenotype of Pep-13 infiltrated (left panel) and wounded leaf tissue (right panel) of wild type (WT), empty vector carrying plants (EV) and *StABCG1*-CRISPR-edited lines 132, 142, 143, 149 and 155. Photographs were taken 10 days after treatment. (**D**) Wounded leaves of wild type and *StABCG1*-CRISPR edited line 143 were stained with SudanIII 7 days after wounding and subjected to microscopy. Visualization of differences in suberin staining of wild type (**E**) and *StABCG1*-CRISPR line 143 (**F**) was performed by 3D plotting of pixels obtained from wound areas according to their RGB values (n = 200).

To address the effects of editing the *StABCG1* gene, transcript levels were determined in Pep-13 infiltrated and wounded leaves of the CRISPR-edited lines. In all five plants, *StABCG1* transcript levels were significantly reduced in response to both treatments compared to control plants (wild type and empty vector containing plants; “C” in Fig. 5A, B), suggesting that transcripts of the edited alleles do not accumulate to high levels. Interestingly, both Pep-13 treatment and wounding resulted in a distinct browning of the tissue adjacent to the Pep-13-induced necrotic tissue or the wound site, respectively (Fig. 5C) in all edited plants. In contrast to wounded tissue of wild type plants, SudanIII-treatment of wounded leaf tissue of *StABCG1*-CRISPR plants did not result in reddish staining (Fig. 5D). Instead, the wound site was bordered by a layer of brownish tissue (Fig. 5D). To visualize the reduced red staining in *StABCG1*-CRISPR plants, the color of selected areas of the SudanIII-stained wounded tissue was determined as RGB values, which were subsequently plotted in a 3D diagram (Fig. 5E, F). These results suggest that editing the *StABCG1* allele results in reduced *StABCG1* transcript levels and reduced suberization of wounded tissue.

### RNA sequence analysis

**Fig. 6:**
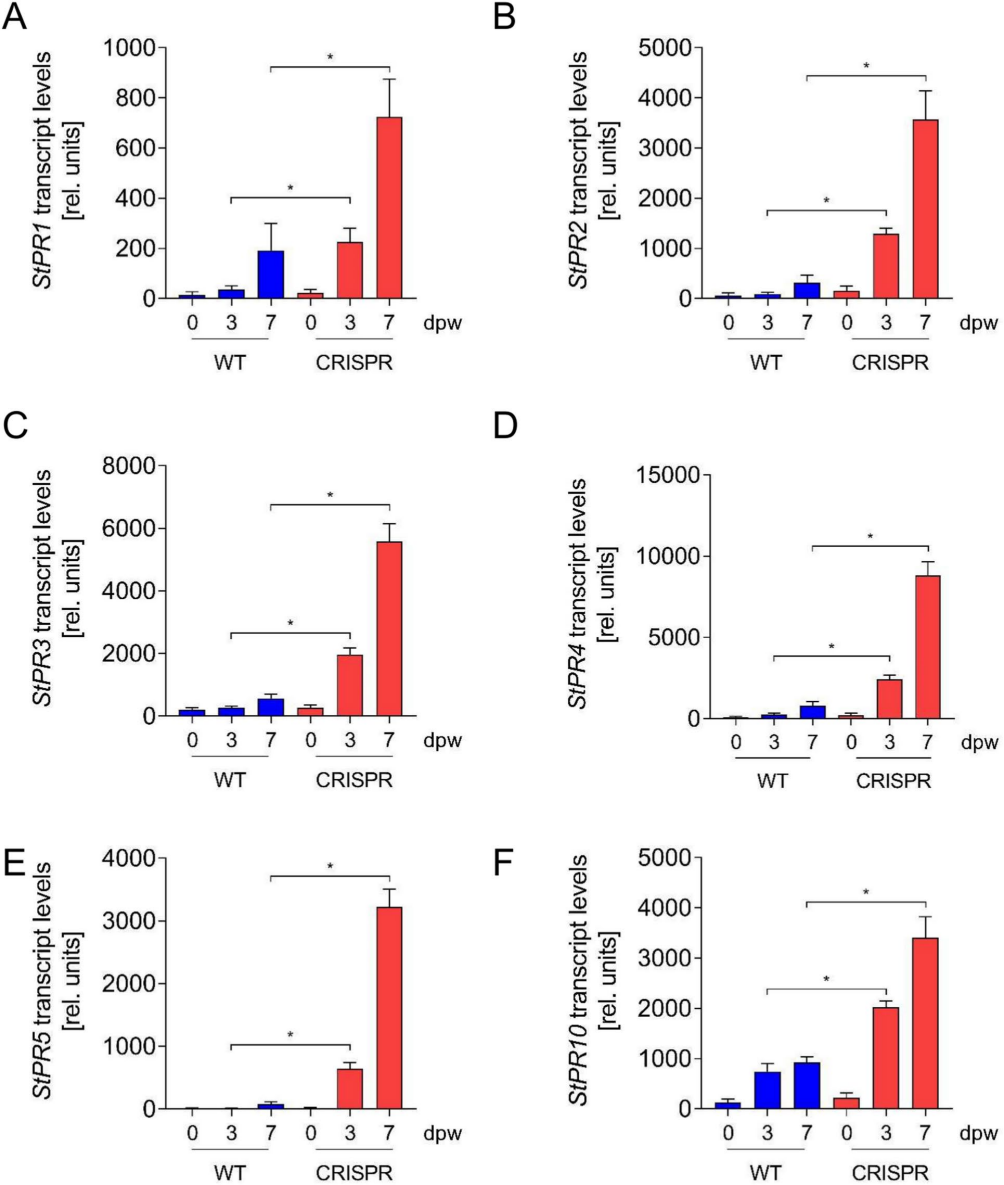
Enhanced *PR* gene expression in *StABCG1*-CRISPR plants. RNA from wounded leaf tissue of wild type (“WT”) or the *StABCG1*-CRISPR plants 132 and 143 (“CRISPR”) was isolated at 0, 3 and 7 days after wounding and sequenced. Data shown are derived from three independent experiments (n = 3 for WT, n = 6 for CRISPR lines). Statistical analyses were performed using Mann Whitney U test (*p < 0.05). PR1: PGSC0003DMG400005113, PR2: PGSC0003DMG400029830, PR3: PGSC0003DMG400001528, PR4: PGSC0003DMG400019437, PR5: PGSC0003DMG400004259, PR10: PGSC0003DMG402001494.

**Fig. 7:**
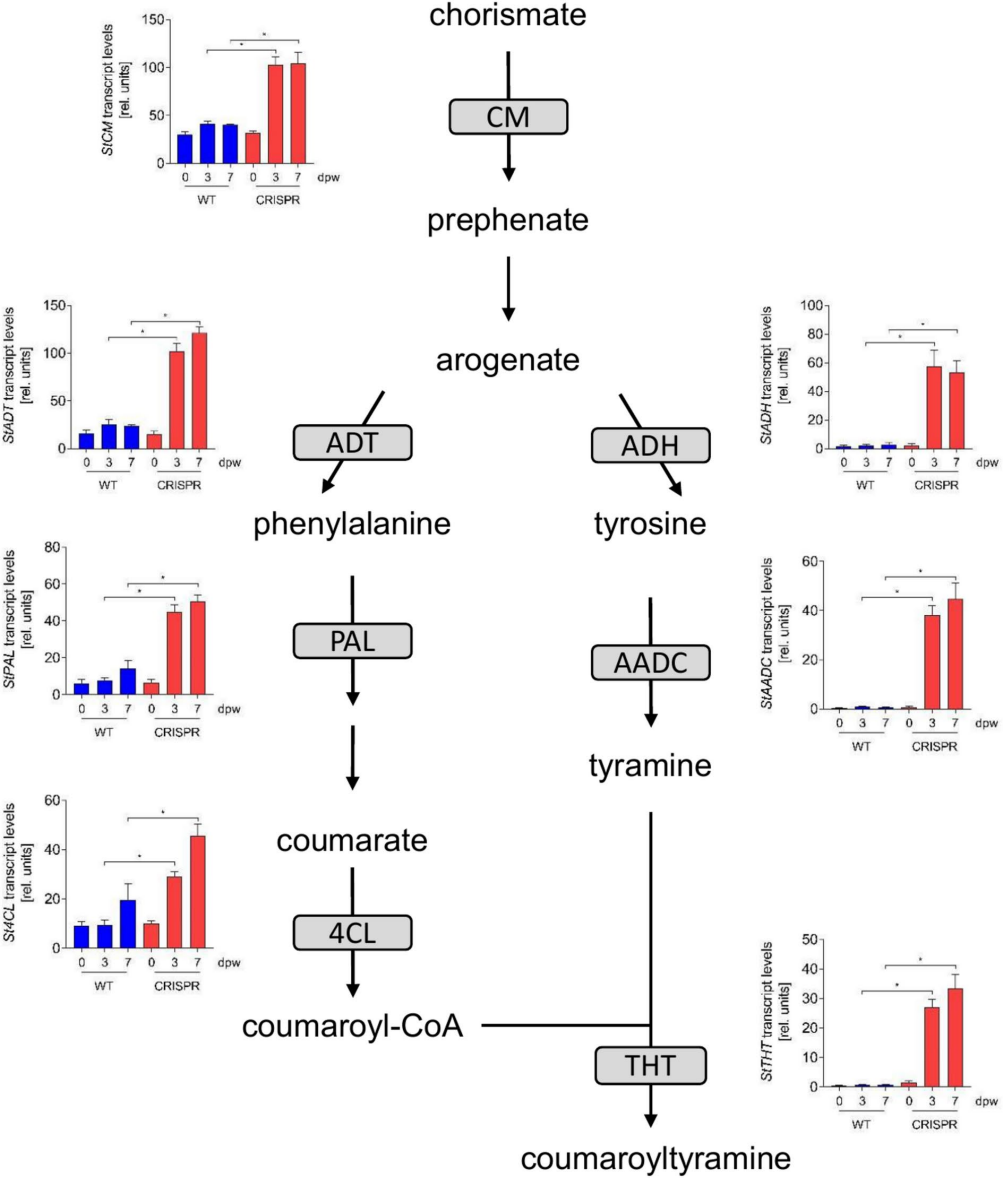
Enhanced expression of genes predicted to encode enzymes of aromatic amino acid and hydroxycinnamic acid amide biosynthesis in *StABCG1*-CRISPR plants. RNA from wounded leaf tissue of wild type (“WT”) or the *StABCG1*-CRISPR plants 132 and 143 (“CRISPR”) was isolated at 0, 3 and 7 days after wounding and subjected to sequencing. Data shown are derived from three independent experiments (n = 3 for WT, n = 6 for CRISPR lines). Statistical analyses were performed using Mann Whitney U test (*p < 0.05). CM: chorismate mutase, PGSC0003DMG400001438; ADT: arogenate dehydratase, PGSC0003DMG400007122; PAL: phenylalanine ammonia lyase, PGSC0003DMG401021549; 4CL: 4-coumarate-CoA ligase, PGSC0003DMG400003155; ADH: arogenate dehydrogenase, PGSC0003DMG400020334; AADC: aromatic amino acid decarboxylase, PGSC0003DMG400014779; THT: tyramine hydroxycinnamoyl transferase, PGSC0003DMG400014778.

To assess differences in gene expression between wild type and *StABCG1*-CRISPR plants upon wounding, leaves were wounded with a corkborer. Wound-adjacent tissue was generated with a larger corkborer 0, 3 and 7 days after initial wounding. The rings thus obtained were used to isolate RNA, which was subsequently subjected to RNA sequencing (Supplemental Table S5). Out of 17,599 genes detected, 599 and 146 genes were activated more than 2.5 fold and tenfold, respectively, three days after wounding in *StABCG1*-CIRSPR plants compared to wild type plants. In contrast, 279 genes were repressed at least 2.5 fold and only six genes were repressed at least tenfold. Within the 599 genes, the highest expressed ones were those predicted to encode pathogenesis-related (PR) proteins, such as antifungal protein PR1, 1,3-β-glucanase (PR2), chitinase (PR3), chitinase type II (PR4), thaumatin (PR5) and PR10 (Fig. 6A–F), in addition to non-specific lipid transfer proteins and proteinase inhibitors. Genes predicted to encode enzymes of aromatic amino acid biosynthesis, such as chorismate mutase (CM), arogenate dehydratase (ADT) and arogenate dehydrogenase (ADH) were significantly higher expressed in *StABCG1*-CRISPR plants (Fig. 7). Moreover, genes encoding enzymes of the phenylpropanoid pathway, i.e. phenylalanine ammonia lyase (PAL) and 4-coumaroyl-CoA ligase (4-CL), as well as aromatic amino acid decarboxylase (AADC) and tyramine hydroxycinnamoyl transferase (THT) were upregulated in *StABCG1*-CRISPR plants (Fig. 7), suggesting a preferential stimulation of the synthesis of aromatic amino acid-derived defense and cell wall reinforcing compounds, such as HCAAs, in suberin-deficient wounded tissue.

### Metabolomics reveal major changes upon wounding of *StABCG1*-CRISPR plants

**Fig. 8:**
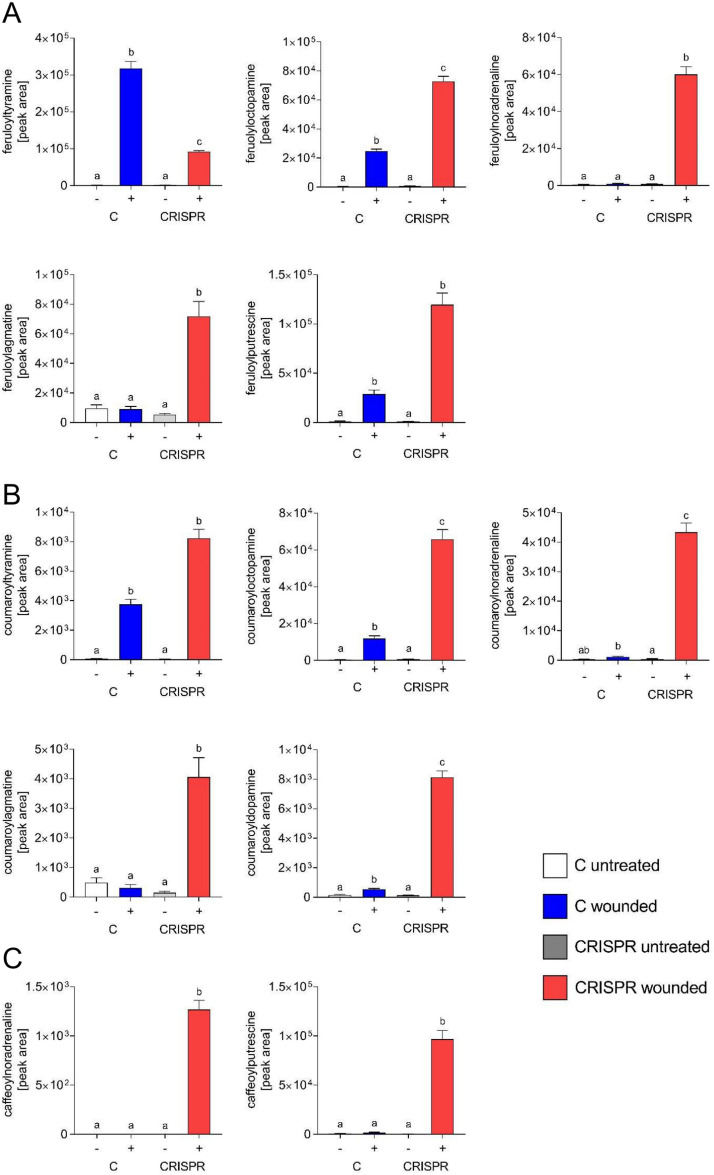
Differentially accumulating metabolites in *StABCG1*-CRISPR plants. Metabolites were extracted from wild type and empty vector carrying potato leaves (“C”), as well as from *StABCG1*-CRISPR plants 132 and 143 (“CRISPR”) either untreated (−) or wounded for ten days (+) and subjected to UPLC-ESI-QTOF-MS analyses. (**A**) Levels of ferulic acid conjugates feruloyltyramine (*m/z* 314.1374, RT 5.9), feruloyloctopamine (*m/z* 330.1335, RT 5.35), feruloylnoradrenaline (*m/z* 328.1179, RT 4.43), feruloylagmatine (*m/z* 307.1753, RT 3.63) and cis-feruloylputrescine (*m/z* 265.1544, RT 3.09). (**B**) Levels of coumaric acid conjugates coumaroyltyramine (*m/z* 284.1283, RT 5.39), coumaroyloctopamine (*m/z* 282.1125, RT 4.58), coumaroylnoradrenaline (*m/z* 298.1067, RT 4.19), coumaroylagmatine (*m/z* 277.1631, RT 3.38) and coumaroyldopamine (*m/z* 300.1228, RT 5.16). (**C**) Levels of caffeic acid conjugates caffeoylnoradrenaline (*m/z* 332.115, RT 3.75) and caffeoylputrescine (*m/z* 251.1389, RT 2.19). Data are derived from three indpendent experiments (n ≥ 20). Statistical analyses were performed using Kruskal–Wallis test.

Untargeted metabolite profiling of wounded leaf tissue by UPLC-ESI-QTOF-MS revealed 360 and 257 features in the positive and negative mode, respectively, with a peak area of at least 2000, which were at least threefold more abundant in wounded *StABCG1*-CRISPR lines than in control leaves). Strikingly, 77 features were present at more than 50-fold higher levels in wounded *StABCG1*-CRISPR plants, 49 of these more than 100 fold. Annotation was performed for hydroxycinnamic acid amides. Levels of feruloyltyramine, the compound that represented the highest accumulating compound in wounded wild type tissue (Fig. 3A), were less abundant in wounded *StABCG1*-CRISPR plants (Fig. 8A). However, seven of the 32 features with the highest abundance in wounded *StABCG1*-CRISPR tissue were identified as HCAAs, such as the feruloylconjugates feruloyloctopamine, -noradrenaline, -agmatine and -putrescine (Fig. 8A). The amount of the tyramine conjugate of coumaric acid, coumaroyltyramine, was not significantly different in control and *StABCG1*-CRISPR plants (Fig. 8B). In contrast, coumaroyloctopamine, -noradrenaline, -agmatine and -dopamine were greatly enhanced in the CRISPR lines. Several of these compounds were hardly detectable in control plants, either wounded or untreated, nor in untreated *StABCG1*-CRISPR lines. For example, the noradrenaline conjugates of ferulic acid, coumaric acid and caffeic acid (Fig. 8C) were 65, 38 and 1000 fold higher in *StABCG1*-CRISPR tissue, respectively. The reduced suberin formation around wound sites thus resulted in a drastically different metabolite pattern with novel HCAAs appearing upon wounding.

## Discussion

### Transcriptional and metabolic changes in potato leaves upon wounding

Upon wounding, plants need to restrict water loss, prevent damage by oxygen and defend against incoming pathogens. Therefore, a transpiration and gas barrier needs to be formed, which prevents exchange of solutes and gases with the environment. This process of wound healing has mostly been analyzed in potato tubers^12^, whereas the leaf´s response to tissue damage is far less studied. Our transcriptomic analyses of the initial response in wounded leaves revealed that, three days after wounding, most of the genes that are at least 100 fold induced are predicted to encode cell wall associated proteins, such as proline-rich proteins, pectin modifying enzymes, peroxidases, laccases and suberin-associated enzymes. These changes would affect cell wall remodeling, which is in accordance with the formation of a closing layer as described for tubers^15^. This early phase of wound healing involves incorporation of phenolics into the cell wall. Therefore, the activation of genes encoding enzymes of the shikimate and phenylpropanoid pathways, which have been also described for wound healing tubers^16,17^, are in accordance with the formation of phenolic-enforced cell walls, representing either the phenolic domain of suberin^2^ or lignin^3^. Our untargeted metabolomic analysis has identified feruloyl tyramine as one of the highest accumulating compounds detected, showing a more than 250 fold enhancement in wounded compared to control leaves (Fig. 3). Thus, the incorporation of HCAAs into cell walls, which has been demonstrated by several groups^14,18,19^, might contribute to reinforcement during closing layer formation in wounded tissue. Interestingly, conjugates of tyramine and octopamine with ferulic acid, the most abundant hydroxycinnamic acid in suberin, were more abundant than the respective coumaric acid or caffeic acid derivatives in wounded potato leaves.

Wounded tissue represents an entry point for pathogens. PR genes are among the highest expressed genes which are at least threefold induced in wounded leaves, suggesting that, in addition to closing layer formation, defense responses are activated. Extensive transcriptomic analyses of wounded tubers also revealed a rapid and strong induction of PR gene expression^17^. The decrease in PR gene expression in wounded tubers at later time points reflects a dynamic transcriptional reprogramming and, moreover, suggests that closing layer formation, i.e. suberization of cells surrounding the wound site, might be sufficient to protect against pathogen attack^17^. Indeed, the deposition of phenolics during wound suberin formation in tubers correlates with resistance upon subsequent infection with *Erwinia carotovora*^20^.

### Reduced suberin formation leads to drastic changes in the response to wounding

Down-regulated suberin formation after wounding of *StABCG1*-edited plants leads to distinctive browning of the tissue adjacent to the wound site. Browning is due to the accumulation of melanin, a group of high molecular weight compounds that originate from oxidized phenols. Non-enzymatic or enzymatic oxidation of phenolics by peroxidases and polyphenol oxidases (PPOs), such as laccases or catechol oxidase^21^, leads to the formation of quinones, which oxidize other polyphenols and polymerize, but also react with a variety of other compounds such as amino acids and proteins. Due to the heterogenous nature of these compounds, it is difficult to identify them by mass spectrometry. Browning-related compounds identified in other plants such as lettuce^22^, pear^23^ or Protea^24^, are not detected in brown tissue of *StABCG1*-edited plants. The presence of melanin in wounded tissue of *StABCG1*-edited plants suggests enhanced access of oxygen, thus supporting the role of suberin as a gas barrier. Similarly, higher oxygen levels are measured in soybean nodules defective in endodermal lipid polyester deposition, due to a mutation in the gene encoding a suberin-related fatty acid reductase^25^.

Most conspicuously, *StABCG1*-edited plants show drastic changes in wound-induced transcript and metabolite abundance. Upregulation of the shikimate pathway, as concluded from our transcriptome analyses, as well as a more than 1000 fold higher abundance of several HCAAs are already detectable three days after wounding. This suggests that closing layer formation is impaired and that incorporation of phenolics into the cell wall compensates for the lack of suberin deposition. However, based on the postulated oxygen-induced tissue browning, the barrier function of suberin is not restored by this strengthening of the cell wall.

Our results therefore show that (i) the wounded plant attempts to compensate for the loss of suberin barrier formation by *de nov*o synthesis of phenolics and (ii) that the accumulation of these phenolics does not results in the formation of an effective barrier.

## Methods

### Plant culture and treatments

Potato plants (*Solanum tuberosum* cv. Désirée, Bioplant Epstorf) were cultivated in sterile tissue culture in a phytochamber (16 h light, ~ 140 µE, 22 °C). Plants were transferred to steam-sterilized soil and grown under long day conditions in a phytochamber (16 h light, ~ 140 µE, 60% humidity, 20 °C). PAMP treatment was performed by infiltration of a 100 µM solution of the oligopeptide Pep-13 or its inactive analog W2A^9^ into the abaxial side of leaves of 3-week-old potato plants growing in a phytochamber. For wounding experiments, leaf disks were cut out with a corkborer. Wounded tissue was obtained by subsequently using a larger corkborer to obtain rings of wounded tissue.

### Suberin staining

Wounded leaf tissue was incubated with a saturated solution of Sudan III for one hour at 70°C and destained by incubation in ethanol. Microscopic evaluation was carried out using a Nikon AZ100 stereomicroscope. To visualize differences in suberin staining, same size areas were scanned from Sudan III-stained microscopic slides. RGB values were determined for each pixel. The mean values for each scanned area were plotted according to their RGB values using Python (https://github.com/python-visualization).

### RNA expression analyses

RNA was isolated from potato leaves using the RNeasy Plant Mini Kit (QIAGEN, Hilden, Germany) and treated with RNase-free DNase Set (Qiagen). cDNA synthesis was performed using the RevertAid H Minus First Strand cDNA Synthesis Kit (ThermoFisher Scientifics, Waltham, USA). Quantitative PCR reactions using EvaGreen were measured on a CFX96TM Real-Time System (Bio-Rad, Hercules, USA). The following primers were used in the amplification: 5′-ATGTCCTCAACTTTGCTAAGAG-3′ and 5′-ACTCCTTCACCTTCTCCAC-3′ for *StFAR1*, 5′-TTGGCTTACCTCCAAATGAAA-3′ and 5′-CGGTAATAGCCGGTAACGAA-3′ for *StCYP86A33*, 5′-TTGACAATTAGCCAGGAAATGA-3′ and 5′-TGCTTCTGCCTCAACAAAAA-3′ for *StFHT*, 5′-GGTCGCGATCCTAGCCTATT-3′ and 5′-ATCCTATCGCGATTGATGAAG-3′ for *StABCG1* and 5′-CACTGCCCAGGTCATCATC-3′, 5′-GTCGAGCACTGGTGCATATC-3′ for *StEF1α*. Relative transcript levels were obtained by standardizing to those of StEF1*α* using the Δct method^26^.

### Transcriptome analyses

Leaves of wild type and *StABCG1*-CRISPR plants 132 and 143 were wounded with a corkborer. Wound-adjacent tissue was generated with a larger corkborer 0, 3 and 7 days after initial wounding. Triplicates of rings thus obtained were immediately shock-frozen in liquid nitrogen. Frozen material was ground under liquid nitrogen conditions. 100 mg of cryo-ground leaf powder was used to isolate total RNA using the RNAeasy kit from QIAGEN and treated with DNase (RQ1, Promega). The quality of total RNA was measured by evaluating RNA integrity number (RIN) using BioAnalyzer quality control analysis (Agilent, Santa Clara, CA) and 1 μg of RNA from samples with a RIN > 8 were sent to NOVOGENE (UK) COMPANY LIMITED for RNA sequencing. The sequenced reads were mapped to the reference genome (solanum_tuberosum_soltub_3_0_gca_000226075_1) using hisat2 (version 2.0.5) with default parameters. The mapped reads were assembled into transcripts using Stringtie (version 1.3.3b), specifically selecting transcripts of class code type ‘u’^27^. Transcripts were quantified using featureCounts (version 1.5.0-p3) with default parameters. Differential expression analysis was performed using DESeq2 (version 1.20.0^28,29^) for samples with biological replicates or edgeR (version 3.22.5) for samples without biological replicates. Genes with, FPKM ≥ 0.5, log2FC (FoldChange) ≥ 1 and adjusted *p* value ≤ 0.05 were considered differentially expressed. Enrichment analysis was performed using clusterProfiler (version 3.8.1) to identify GO terms and KEGG pathways that were enriched among the differentially expressed genes, with a significance threshold of padj < 0.05. Gene Set Enrichment Analysis (GSEA) was performed using gsea (version v3.0). A threshold of a log2FC ratio ≥ 1 was used to define significant DEG after cutting off the data at a false discovery rate (FDR) value ≤ 0.05 for all unigenes with $\ge$ 0.5 FPKM. Functional enrichment analysis and protein–protein interaction analysis were also performed.usegalaxy.eu^30^. The raw data for RNA sequencing and sampling details are deposited at the NCBI as Bioproject PRJNA1132763. By use of MAPMAN 4.0 we further categorized all detectable transcripts and performed k-means time cluster analysis^31^. Also in parallel, Gene ontology (GO) enrichment analysis was conducted by DAVID V. 6.8^32,33^.

### Metabolomic analyses

Rings of wounded leaf tissue obtained as described above was used for metabolomic analyses. Chromatographic separations of methanolic extracts were performed as described previously^34,35^ with the following modifications of the binary gradient with 0.150 µl/min: 0 to 1 min isocratic 95% A (water/formic acid, 99.9/0.1 [v/v]) and 5% B (acetonitrile/formic acid, 99.9/0.1 [v/v]); 1 to 10 min linear from 5 to 60% B; 10 to 10.2 min linear to 95% B; 10.2 to 12 min isocratic 95% B; 12 to 14 min isocratic 5% B. Eluting compounds were detected from m/z 50 to 1000. LC–MS profiling data were analysed as described previously^35,36^.

### Generation of CRISPR-edited potato plants

The position of the guide RNA was chosen as a highly conserved region of the cDNA and the potato genome sequence (*S. tuberosum* group phureja; PGSC) encoding the nucleotide binding domain of the ABC transporter. The guide RNA was synthesized and cloned under the control of an Arabidopsis U6 promoter^37^. The CRISPR construct pAGM31059 was cloned using the modular cloning system MoClo into the level 2 binary vector pAGM4723^38^, which contains a kanamycin cassette for selection of transformants (level 1 module pICH67131), a Cas9 expression cassette and a guide RNA cassette. The Cas9 expression cassette contains a *Zea mays* codon-optimized Cas9 coding sequence that contains 13 Arabidopsis introns for high level expression in plants^39^. The Cas9 coding sequence (level 0 module pAGM13741) contains a C-terminal NLS and was cloned under control of the 35S promoter with the Ocs terminator.

*Agrobacterium tumefaciens*-mediated leaf disk transformation was performed as reported^40^. Transgenic plants were subjected to PCR analyses to detect edited alleles using the primers 5′-CTCCTTCAACAACCTCACCTACAG-3′ and 5′-GTGGCAATCTGAATTCAGCTGCAA-3′, covering the guide RNA target site 5′-GGCGAGATAGTCGCCGTCCT-3′. Illumina sequencing of PCR products was performed by Novogene (en.novogene.com/).

### Accession numbers

CM: chorismate mutase, PGSC0003DMG400001438; ADT: arogenate dehydratase, PGSC0003DMG400007122; PAL: phenylalanine ammonia lyase, PGSC0003DMG401021549; 4CL: 4-coumarate-CoA ligase, PGSC0003DMG400003155; ADH: arogenate dehydrogenase, PGSC0003DMG400020334; AADC: aromatic amino acid decarboxylase, PGSC0003DMG400014779; THT: tyramine hydroxycinnamoyl transferase, PGSC0003DMG400014778; PR1: PGSC0003DMG400005113, PR2: PGSC0003DMG400029830, PR3: PGSC0003DMG400001528, PR4: PGSC0003DMG400019437, PR5: PGSC0003DMG400004259, PR10: PGSC0003DMG402001494.

## Supplementary Information

Supplementary Tables.

Supplementary Table S5.

Supplementary Legends.

## Data Availability

The Metabolomics raw and processed data have been submitted to the EMBL-EBI MetaboLights repository as accession MTBLS4823, and are accessible under https://www.ebi.ac.uk/metabolights/MTBLS4823. The RNA-seq data have been submitted to the SRA database under accession PRJNA1132763, and are accessible at https://www.ncbi.nlm.nih.gov/sra/PRJNA1132763.
