# Supplementary material for: The suberin transporter StABCG1 is required for barrier formation in potato leaves
Source: Sci Rep. 2025 Mar 7;15:7930. doi: 10.1038/s41598-025-89032-x (PMC11885807; doi:10.1038/s41598-025-89032-x)
Supplement: Supplementary file 1 — Supplementary Tables. [file 41598_2025_89032_MOESM1_ESM.pptx]

## Slide 1
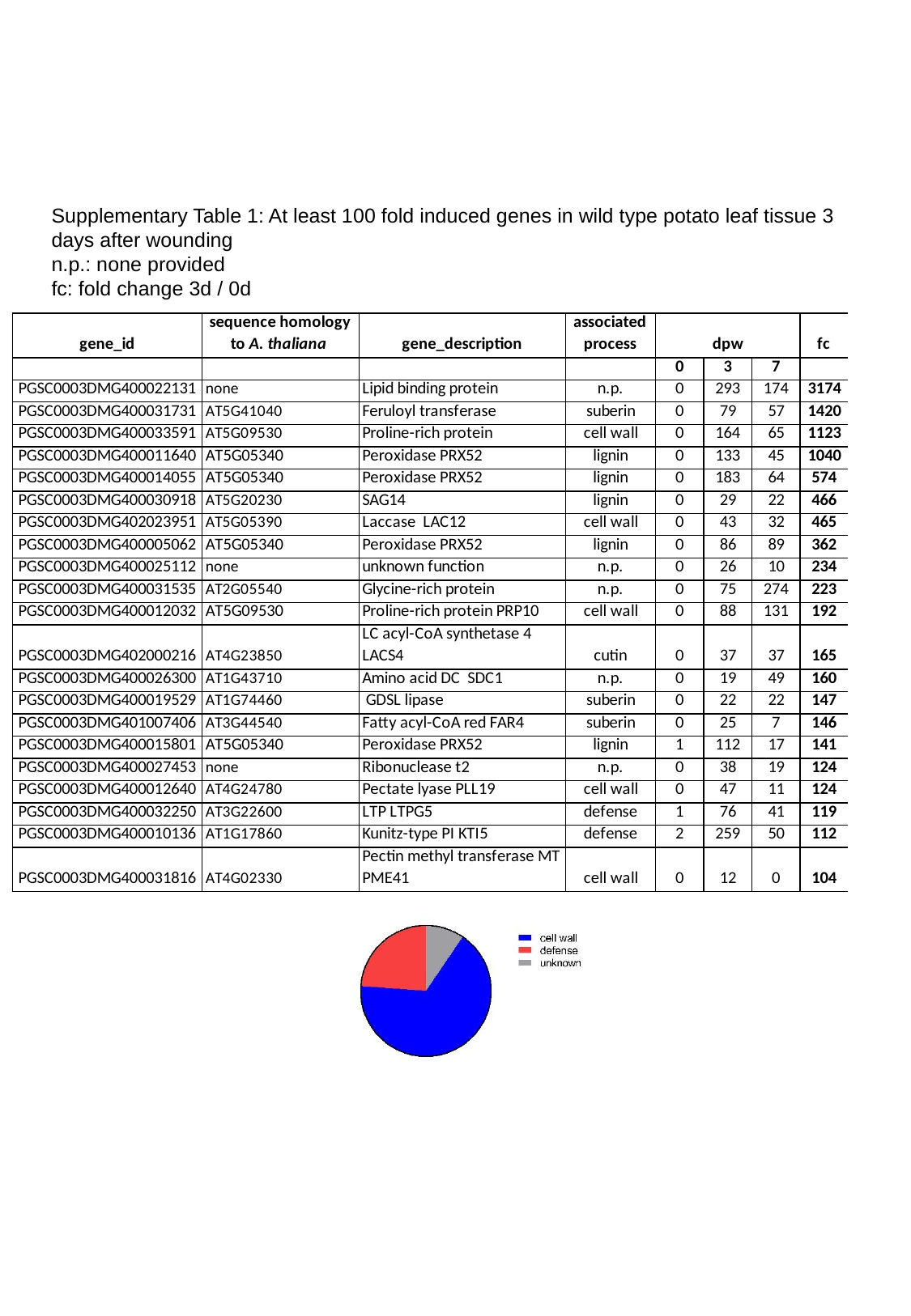

Supplementary Table 1: At least 100 fold induced genes in wild type potato leaf tissue 3 days after wounding
n.p.: none provided
fc: fold change 3d / 0d

## Slide 2
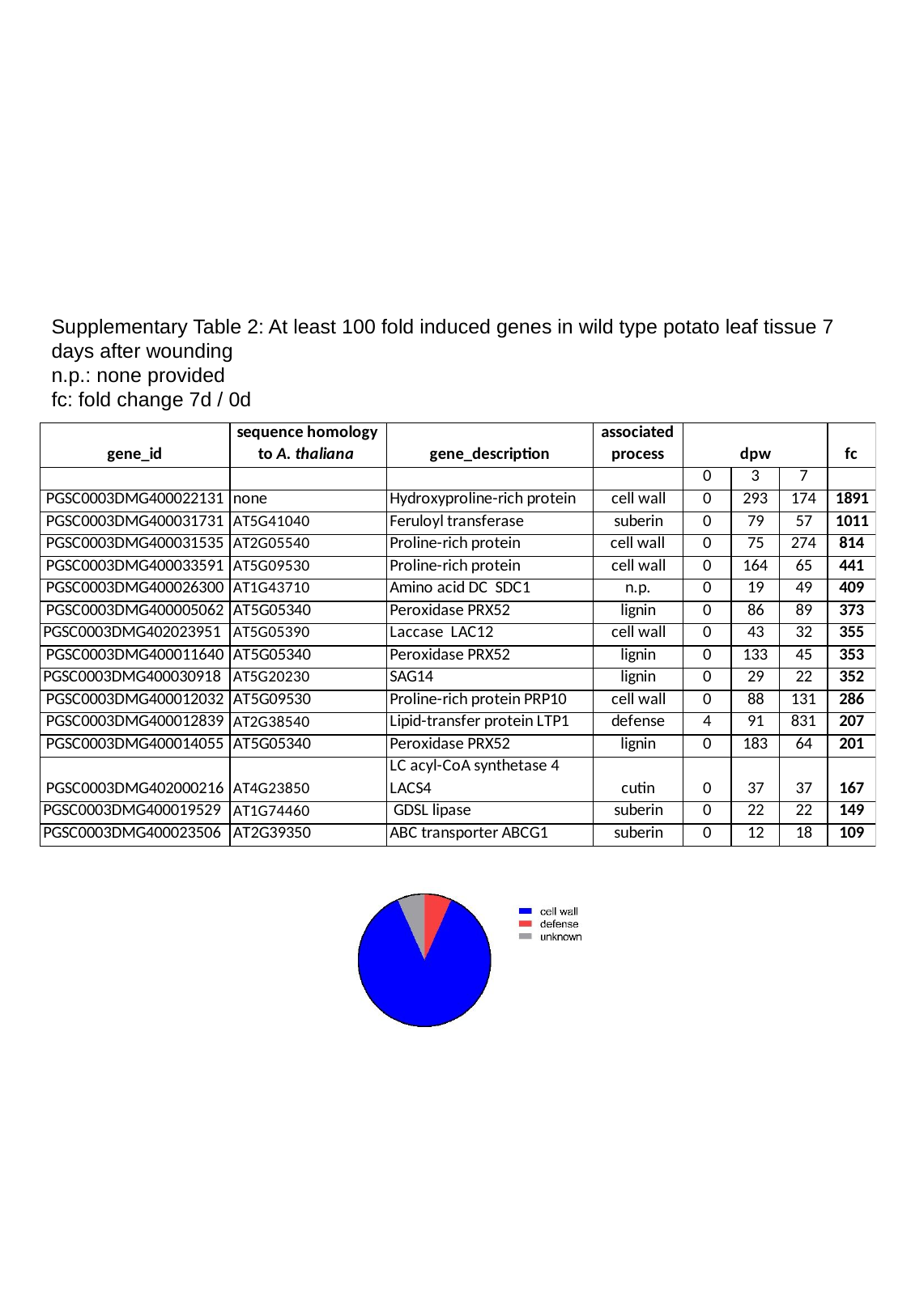

Supplementary Table 2: At least 100 fold induced genes in wild type potato leaf tissue 7 days after wounding
n.p.: none provided
fc: fold change 7d / 0d

## Slide 3
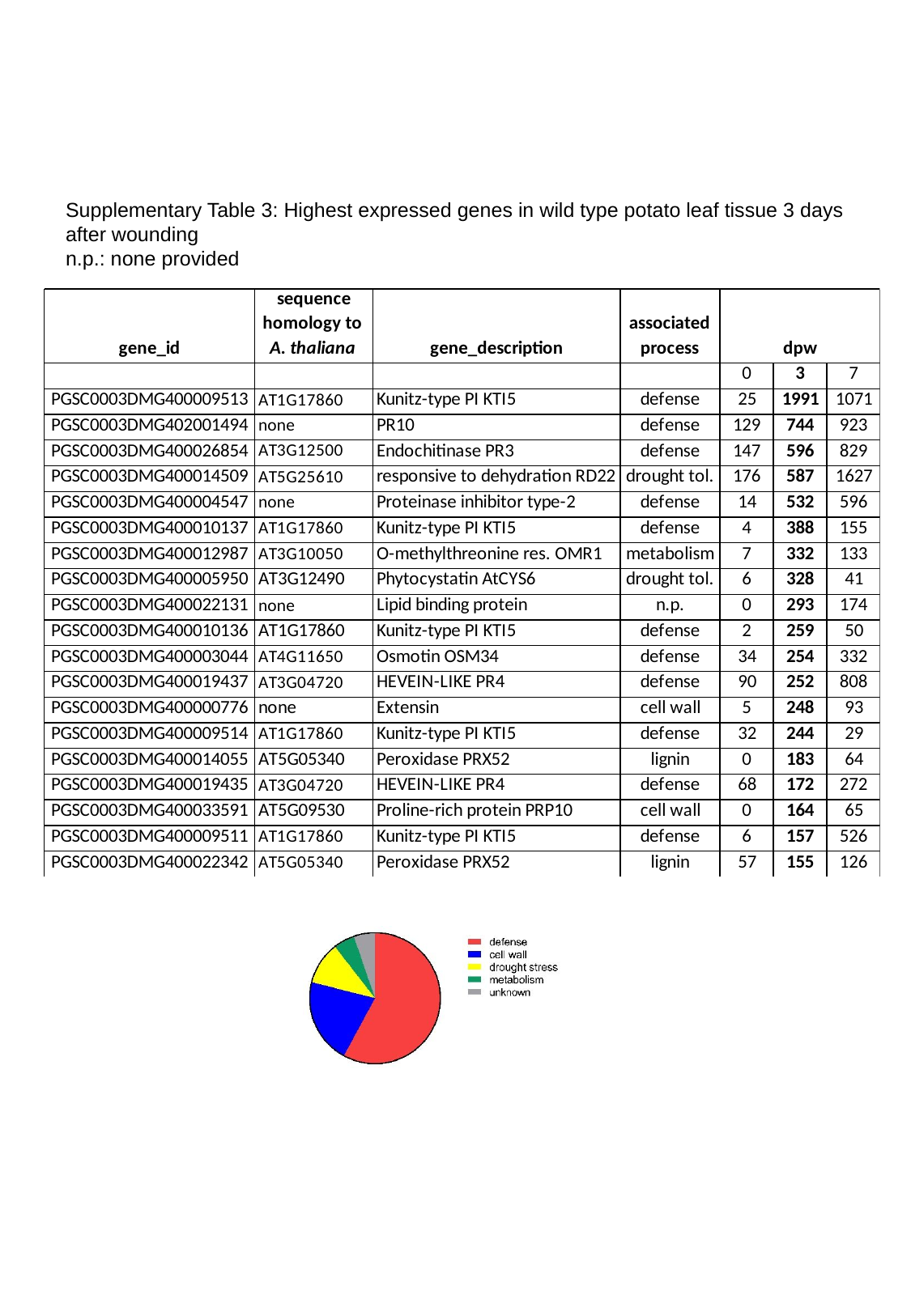

Supplementary Table 3: Highest expressed genes in wild type potato leaf tissue 3 days after wounding
n.p.: none provided

## Slide 4
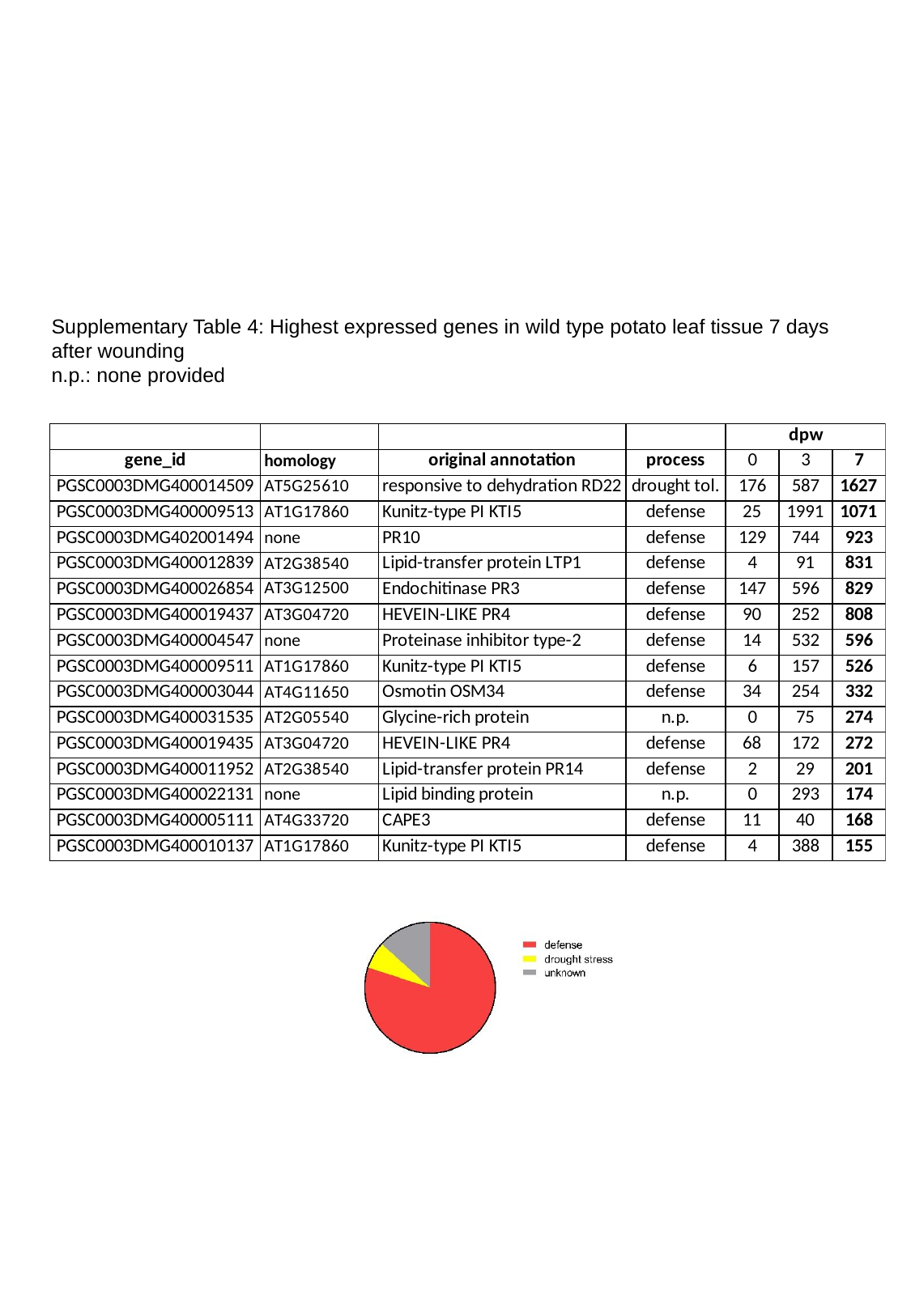

Supplementary Table 4: Highest expressed genes in wild type potato leaf tissue 7 days after wounding
n.p.: none provided
