# Supplementary material for: The suberin transporter StABCG1 is required for barrier formation in potato leaves
Source: Sci Rep. 2025 Mar 7;15:7930. doi: 10.1038/s41598-025-89032-x (PMC11885807; doi:10.1038/s41598-025-89032-x)
Supplement: Supplementary file 3 — Supplementary Legends. [file 41598_2025_89032_MOESM3_ESM.docx]

**Legends for Supplementary Data**

Supplementary Table 1: At least 100 fold induced genes in wild type potato leaf tissue 3 days after wounding

Supplementary Table 2: At least 100 fold induced genes in wild type potato leaf tissue 7 days after wounding

Supplementary Table 3: Highest expressed genes in wild type potato leaf tissue 3 days after wounding

Supplementary Table 4: Highest expressed genes in wild type potato leaf tissue 7 days after wounding

Supplementary Table 5: Enrichment analysis
